# Genetic polymorphisms in vitamin D pathway influence 25(OH)D levels and are associated with atopy and asthma

**DOI:** 10.1186/s13223-020-00460-y

**Published:** 2020-07-09

**Authors:** Alana Alcântara Galvão, Flávia de Araújo Sena, Emília Maria Medeiros de Andrade Belitardo, Maria Borges Rabelo de Santana, Gustavo Nunes de Oliveira Costa, Álvaro Augusto Cruz, Maurício Lima Barreto, Ryan dos Santos Costa, Neuza Maria Alcantara-Neves, Camila Alexandrina Figueiredo

**Affiliations:** 1 Instituto de Ciências da Saúde, Universidade Federal da Bahia (UFBA), Salvador, Bahia, Brazil; 2 Programa de Pós Graduação em Imunologia (PPGIm), Bahia, Brazil; 3 Instituto de Saúde Coletiva, Universidade Federal da Bahia (UFBA), Salvador, Bahia, Brazil; 4 Universidade Salvador (UNIFACS), Salvador, Bahia, Brazil; 5 ProAR, Faculdade de Medicina, Universidade Federal da Bahia (UFBA), Salvador, Bahia, Brazil; 6 Centro de Integração de dados e Conhecimentos para Saúde(CIDACS), Fiocruz, Bahia Brazil; 7 Departamento de Ciências da Biorregulação, Instituto de Ciências da Saúde, Universidade Federal da Bahia, Canela, CEP 41110-100 Salvador, BA Brazil

**Keywords:** Vitamin D, CYP2R1, VDR, CYP24A1, IgE, 25(OH)D, SNVs, Asthma, Atopy

## Abstract

**Background:**

Vitamin D deficiency or insufficiency, has been associated with atopy and lack of asthma control. Our objective was to investigate associations between variants in genes of vitamin D pathway with serum levels of 25-hydroxyvitamin D (25(OH)D), atopy, asthma and asthma severity in teenagers from Northeast Brazil.

**Methods:**

This is a cross sectional study nested in a cohort population of asthma. 25(OH)D was quantified from 968 of 11–17 years old individuals by ELISA. Asthma diagnosis was obtained by using the ISAAC Phase III questionnaire. Specific IgE was determined by ImmunoCAP; genotyping was performed using the 2.5 HumanOmni Biochip from Illumina. Statistical analyses were performed in PLINK 1.07 and SPSS 22.1.

**Results:**

After quality control, 104 Single Nucleotides Variants (SNVs) in vitamin D pathway genes, typed in 792 individuals, were included in the analysis. The allele A of rs10875694 on *VDR* was positively associated with atopy (OR = 1.35; 95% CI 1.01–1.81). The allele C of rs9279 on *VDR*, was negatively associated with asthma risk (OR = 0.66; 95% CI 0.45–0.97), vitamin D insufficiency (OR = 0.78; 95% CI 0.70–0.96) and higher VDR expression. Two variants in *VDR* were associated with asthma severity, the allele A of rs2189480 (OR = 0.34; 95% CI 0.13–0.89) and the allele G of rs4328262 (OR = 3.18; 95% CI 1.09–9.28). The combination of variants in *CYP2R1* and *CYP24A1* (GAC, to rs10500804, rs12794714 and rs3886163, respectively) was negatively associated with vitamin D production (*β* = − 1.24; 95% CI − 2.42 to − 0.06).

**Conclusions:**

Genetic variants in the vitamin D pathway affect vitamin D serum levels and, thus, atopy and asthma.

## Background

Asthma affects more than 339 million people worldwide and it is estimated leading to the death of almost 400,000 people by year [1, 2]. This disease is characterized by a chronic inflammation of lower airways that include complex pathophysiological mechanisms involving several pro-inflammatory cells and molecules, including different cytokine profiles that can change according to environmental and genetic factors [3, 4]. Asthma immunopathological processes leads to reversible airflow obstruction, increased mucus secretion and airway remodelling. Allergic (or atopic) asthma is characterized by the presence of ILC2 and T helper 2 (Th2) response that covers the production of cytokines such as, interleukin (IL)-4, IL-5 and IL-13 which all together orchestrate the migration of eosinophils, mast cells activation and Immunoglobulin E (IgE) production [5, 6]. Subjects with asthma may be atopic or nonatopic. Those with Type 2 inflammation, likewise can also be nonatopic [7]. Atopy is an inherited predisposition to produce IgE in response to exposure to allergens, such as house dust mites, pollen, fungi and food proteins. Atopic individuals can present dermatitis, rhinitis, asthma or can be asymptomatic [8]. In addition to that, all the other asthma phenotypes that do not include sIgE production (specific Immunoglobulin E) are classified as non-atopic [3]. However, asthma heterogeneity involve many different immunological mechanisms and there are likely overlaps among them [9]. Previous studies from our group in the same population of the current study have shown that 24,5% of asthma cases were attributed to atopy [10] and that IFN-γ could be an important biomarker of non-atopic asthma in this population [11]. The main risk factors for this asthma phenotype include poverty and dirt conditions [12]. These findings suggest that asthma in Latin America could differ from Europe and other developed countries [13].

Beyond its role in mineral bone regulation, vitamin D also has a key role in immune regulation [14]. Vitamin D is the general term for a group of secosteroid metabolites whose active form is 1α-25-dihydroxyvitamin D (1,25(OH)D) [15]. This hormone is involved in the regulation of several immune cells, such as lymphocyte, macrophage, monocyte and eosinophils [14] and immune biomarkers, such as CD86/80, FOXP3, MHC, cytokines and IgE [14, 16–18]. The regulation of immune system occurs with vitamin D receptor (VDR) binding on elements responsive to vitamin D (VDRE) on many target genes of immune cells, determining their transcription or silencing [19]. Thus, this molecule has been shown to be a protective factor to diverse immunopathologies such as diabetes type I, multiple sclerosis, psoriasis, allergies and asthma [15, 20–22].

Studies have shown that low serum levels of vitamin D are associated with asthma risk and reduced forced expiratory capacity in one second (FEV1) as well as forced vital capacity (FVC)  [23–25]. Moreover, some studies assert that supplementation of asthmatic children with vitamin D resulted in an improvement of pulmonary function, prevention of asthma exacerbation and reduction of IgE sensitization [26–29], although there are controversial findings in the literature [30–33]. Otherwise, maternal intake of vitamin D during pregnancy has been correlated with lower asthma diagnostic in offsprings [34].

**Fig. 1:**
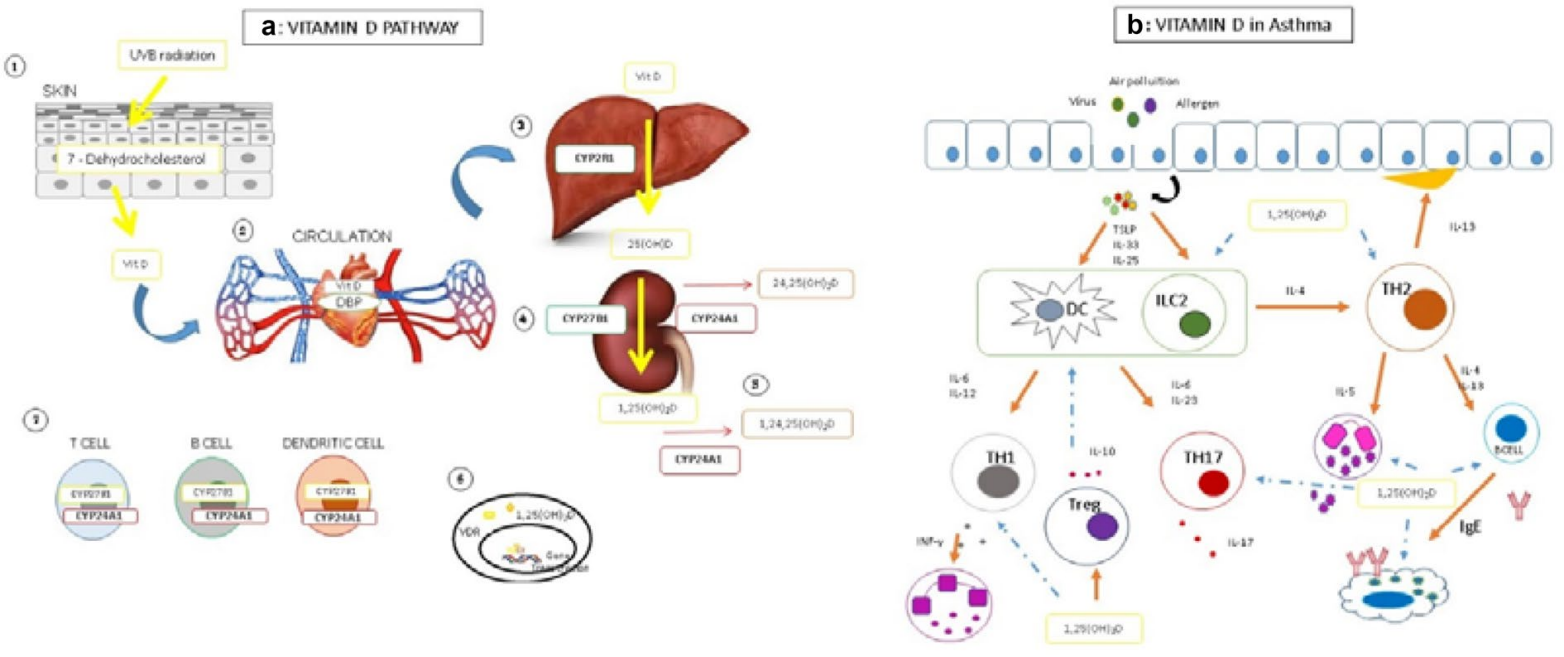
Vitamin D pathway and immunological activity.**a** Vitamin D pathway. 1—Vitamin D starting your synthesis in skin by UVB radiation; 7-dehydrol cholesterol is converted to pre vitamin D3 by UVB radiation. 2—A subsequent thermal isomerization form vitamin D3, that reaches bloodstream and binds to DBP being transported to the tissues. 3—In the liver it is metabolized to 25-hydroxyvitamin D3 by CYP2R1, 4—following another hydroxylation occurs in kidney by CYP27B1 to form 1,25 dihydroxyvitamin D3. The vitamin D levels are regulated by a feedback mechanism over CYP24A1 that hydroxylates 1,25(OH)_2_D and/or 25(OH)D in position 24, generating an inactive metabolite. 5—Immune cells express CYP27B1 and CYP24A1, being able to regulate vitamin D paracrine metabolism. 6—1,25(OH)_2_D binds to VDR and regulates gene expression. **b** Vitamin D in asthma. Asthma can be triggered by many factors such virus, allergens and air pollution; allergens frequently lead epithelial cells to release cytokines IL-25, IL-33 and TSLP, that lead to a Th2 maturation, including ILC2 activation. Th2 cells release Il-4, IL-5 and IL-13 that lead to mast cells and eosinophils recruitment and IgE production that leads to mast cell degranulation and amplification of inflammation. IL-13 leads to increase mucus production in epithelium. The inflammatory cell infiltration leads smooth muscle thickening and contraction; reduction of airway lumen culminates to induce asthma symptoms. A non Th2 asthma profile, can be trigged by virus and air Pollutants, and lead to a Th1 or Th17 response, of neutrophilic profile. T reg cells reduce Th1, Th2 and Th17 response controlling inflammation. 1,25(OH)D can inhibit ILC2, eosinophilic and mast cell proliferation; Inhibits IL-4 release and IgE synthesis. 1.25(OH)D induces Treg response. Orange arrow—activation. Blue arrow—inhibition. *TSLP* thymic stromal lymphoprotein

The synthesis of active vitamin D includes reactions that occur in three different tissues [35]. The initial production occurs in the skin, by conversion of 7-dehydrocholesterol following UV irradiation to Vitamin D_3_. Vitamin D_3_ is transported in blood circulation by DPB (Vitamin D Protein Binding) and in the liver it is metabolized to 25 hydroxyvitamin D_3_ (25(OH)D) by CYP2R1; in the kidney it undergoes other hydroxylation, by CYP27B1, leading to the active form 1,25(OH)_2_D. It is worth highlighting that immune cells also present CYP27B1 [14]. The 1,25(OH)_2_D binds to the VDR (Vitamin D receptor), a nuclear receptor that regulates target gene transcription. The vitamin D levels are regulated by a feedback mechanism over CYP24A1 that hydroxylates 1,25(OH)_2_D and/or 25(OH)D in position 24, generating an inactive metabolite [15]. Nevertheless, there are observations about the function of the “inactive” metabolite 24,25 dihydroxyvitamin D, in bone metabolism [36]. Figure 1 shows vitamin D pathway and vitamin D possible effect in asthma immunopathology.

Genetic studies have contributed to identifying the molecular pathways that affects asthma [37, 38]. A recent review showing a survey of 10 years of genome-wide studies of asthma, highlighted 28 main genes involved in asthma with reproducible data. Some of these genes are involved in immune function and were related with asthma or other allergies, including *TSLP, TNFSF4, CD247, GATA*-*3, RORA, TLR1, IL6R* and *IL2RB* [39]. Once vitamin D is an important modulator of immune system response and *VDR* is a map to chromosome 12q, near a region linked to asthma, variants in genes from vitamin D pathway can affect asthma and atopy. Variants in and vitamin D pathway gene (*VDR, CYP2R1, CYP24A1, CYP27B1, DBP)* are highlighted in immunopathologies including allergies, particularly in asthma [40–48].

Preliminary observation of ours indicate insufficient vitamin D levels were found in 60% of adolescents of the city of Salvador, in the Northeast of Brazil of tropical climate with an average yearly temperature of 27 °C and estimated radiation of 5.9 kWh/m^2^ [49]. Similar observations were reported in a population of South-eastern Brazil [50]. To explore this paradox, we hypothesized that genetic variants in vitamin D pathway genes may affect 25(OH)D levels and then may affect atopy and asthma in the Brazilian population.

## Materials and methods

### Population and study design

This work was conducted in the SCAALA Cohort (Social Change Asthma and Allergy in Latin America, Salvador, Bahia, Brazil) [51]. The study design has been described previously [52, 53]. Briefly, the original study population was composed by 1445 children, living in 24 deprived areas from the city of Salvador, Northeast Brazil, enrolled in the study to evaluate the impact of a sanitation programme on diarrhoea occurrence over the period from 1997 to 2003, when the participants were 0–3 years old [54]. The first survey on risk factors for wheezing and atopy was conducted in 2005, the second in 2007 and the third in 2013 [10, 52, 55]. The information for the present study were obtained in 2013 when data from 1206 participants were collected. At this time, the population aged 11 to 19 years old. Seven hundred and ninety-two individuals with complete data of interest were included in the current analysis. In addition, the 25(OH)D levels and specific IgE for aeroallergens (sIgE) were quantified, and vitamin D pathway genes were typed for variants. Asthma diagnosis was obtained using the International Study of Asthma and Allergy in Childhood phase III questionnaire (ISAAC PHASE III) adapted to Portuguese, answered by parents or legal guardians of each participant, or the participant himself if 18 years or older.

Ethical approval was obtained through the Ethical Committee for Health Research of the Institute of Public Health of the Federal University of Bahia, Brazil (Num. 120.616). Written informed consent was obtained from the legal guardian of each individual if they were under 18 years, and themselves if they were older than 18 years old.

### Asthma definition

Asthma was defined as wheezing in the last 12 months, plus at least one of the following: (1) history of asthma ever, (2) 4 or more wheezing episodes in the last 12 months, (3) wheezing with exercise in the last 12 months, and (4) sleep disorder due to wheezing in the last 12 months [55]. All other individuals were classified as non-asthmatics.

The asthma severity was also obtained by ISAAC questionnaire as previously described [55]. Severe asthma, from the epidemiological stand point, was defined as individuals having at least one of the following symptoms in the last 12 months: (1) ≥ 12 wheezing episodes, (2) wheezing and breathlessness resulting in difficulty in speaking, and (3) > 1 day of disturbed sleep/week due to asthma. The other cases were considered as mild/moderate asthma.

### Atopy definition

Previous study in our population, have indicated that the prevalence of allergen-specific IgE (sIgE) for the studied aeroallergens was greater than the skin prick test (SPT) positivity, and the frequency of SPT positivity between those without sIgE was very low [56]. For this reason, atopy was defined as the presence of at least one positive test for a relevant aeroallergen with sIgE ≥ 0.70 kU/L. The sIgE was determined by ImmunoCap using caps to *Blomia tropicalis, Dermatophagoides pteronyssinus, Blatela germanica,* and *Periplaneta americana* from Phadia (AB, Uppsala Sweden).

### 25-hydroxy vitamin D serum levels quantification

An inhibitory enzyme immunosorbent assay (IDS OCTEIA EIA, IDS Bolton, UK) was used to quantify serum levels of 25(OH)D. This is a diagnostic method recognized by the Vitamin D External Quality Assurance Survey (DEQAS). The lower detection limit was 2 ng/mL. Intra-assay and inter-assay coefficients of variation for concentrations between 15.6 and 52.8 ng/mL were < 5.9% and < 6.6%, respectively.

There is no consensus so far for vitamin D deficiency or insufficiency categorization, some authors define deficiency as < 12 ng/mL and insufficiency < 20 ng/mL [57], and others deficiency < 20 ng/mL and insufficiency < 30 ng/mL [58]. We use vitamin D classification as follows: (1) deficient (< 20 ng/mL); (2) insufficient (≥ 20–30 ng/mL) and (3) sufficient (≥ 30 ng/mL). To carry out logistic regression analyses, we used dichotomous variables to define 25(OH)D serum levels using two different cut-offs, first 20 ng/mL(deficiency), second 30 ng/mL (Deficiency + Insufficiency) [43, 58, 59].

### Genotyping and quality control

DNA was extracted from peripheral blood using a commercial kit (Gentra Purgene Blood Kit (Qiagen, Gemantown, ML USA). Genotyping tests were developed using Illumina Human Omni 2.5 BeadChip (San Diego, CA, USA). Five genes in the vitamin D pathway were used in this study, *VDR, CYP2R1, CYP27B1, CYP24A1 and CG/DBP*. The VDR genetic information was extracted from 48,235,320 to 48,298,814 (Location: NC_000012.12) position at chromosome 12; *CYP2R1* information was extracted from 1,489,951 to 14,913,874 (location: NC_000011.10) position at chromosome 11; *CYP24A1* information was extracted from 52,769,985 to 52,790,516 (location: NC 000,020.11) position at chromosome 20; *CYP27B1* information was extracted from 58,156,117 to 58,160,976 (location: NC 000012.12) position at chromosome 12 and *DBP* information was extracted from 49,133,817 to 49,140,639 (location: NC000019.10) position at chromosome 19. Quality control was carried out in PLINK version 1.07. SNVs were excluded if MAF (minor allele frequency) was less than 1%, imbalance of Hardy–Weinberg equilibrium with *P* value less than 10^−4^ and percentage of missing *loci* more than 1%.

### In silico analysis

To analyse genetic expression, an online browser of the Genotype Tissue Expression Project (GTEx) was used (http://www.gtexportal.org). This project established a database which contains tissue gene expression according to the genetic variation. We examined whether genotypes of two *VDR* SNV’s, rs9729 and rs731236, were associated with differential expression of VDR receptor in whole blood.

### Statistical analysis

The statistical analysis for genetics associations between polymorphisms in vitamin D pathway (*VDR, CYP2R1, CYP24A1, CYP27B1* and *DBP)* and asthma, atopy and Vitamin D were performed using PLINK 1.07. Logistic regression was done to estimate ORs and 95% confidence intervals to categorical variables. Linear regression was made to estimate *Beta* and 95% confidence intervals to continuous variables. For such analyses, we used covariates (sex, age, and individual ancestry estimated although 269 informative markers identified to principal component analyses compound two variables PC1 and PC2) [37]. The additive genetic model was applied in these analyses and adaptive permutations were employed to the multivariate analysis. To evaluate the combined effect of SNV’s on *CYP24A1* and *CYP2R1* in 25(OH)D serum levels, and *VDR* SNV’s effect in atopy, asthma and 25(OH)D serum levels, we performed genetic risk score analysis using SNPstats platform (http://www.snpstats.net/start.htm).

The LD plot was done using the Haploview software. The statistical analysis for serum 25(OH)D and SNV’s rs12794714, rs10500804 and rs3886163 were performed using the GRAPHPAD Prisma 7 software (GraphPad Software, San Diego, CA, USA), using Kruskal–Wallis and Dunn’s post-test. We considered as significant associations, those with P-values ≤ 0.05.

## Results

### Description of population

We assessed 942 individuals with blood sample, of these 821 were genotyped and 792 remained in the analyses after the genetics quality control tests. The descriptive data of the studied population are shown in Table 1. The asthmatics individuals correspond to 63 (7.9%) and atopic 364 (45.9%). Four hundred and fifteen (52.4%) were males while 377 (47.6%) were female. Males were significantly more atopic than females (p < 0.001), and younger age [11–14] was significantly more frequent among asthmatics (p = 0,032). About vitamin D status, 165 (20.8%) were deficient, 322 (40.7%) were insufficient and 305 (38.5%) had sufficient levels. No association between vitamin D and atopy or asthma was observed. However, considering the whole population studied (942), vitamin D deficiency was associated with atopy and insufficiency was associated with asthma only among females (data on submission).

**Table 1:** Characteristic of 792 studied subjects

	Asthma n/N (%)	*p value	Atopy n/N (%)	*p value
Variables	*63/792 (7.9)*		*364/792 (45.9)*	
Gender				
Males	30/415 (7.2)	0.434	216/415 (52.0)	*< 0.001*
Female	33/377 (8.8)		148/377 (39.3)	
Age				
11–14	46/478 (9.8)	*0.032*	218/478 (45.6)	0.927
15–19	17/314 (5.4)		146/314 (46.5)	
Vitamin D (ng/mL)				
m ± SD^a^	27.95 ± 8.98/27.33 ± 9.60	0.935	26.98 ± 10.12/27.92 ± 9.01	0.156
Vitamin D levels				
Sufficient	25/305 (8.2)	0.893	131/305 (43.0)	0.188
Insufficiency/deficiency	38/487 (7.9)		233/487 (47.8)	
Vitamin D levels				
Sufficiency/insufficiency	52/627 (8.3)	0.627	277/627 (44.2)	0.054
Deficient	11/165 (6.7)		87/166 (52.7)	

* Mean Whitney test; Numbers in italics are statically significant

^a^*m ± SD* median and standard deviation

### Description of genetic data

After quality control for SNVs and individuals, 104 SNVs in vitamin D pathway genes [*DBP* [2]; *CYP2R1* [4]; *CYP24A1* [37]; *CYP27B1* [1] and *VDR* [59] ] were included in this study in 792 studied individuals. We have identified 20 genetic variants associated with at least one of the studied outcomes (Additional file 1: Table S1).

### *VDR* SNVs are associated with atopy, asthma and asthma severity

Table 2 summarizes the significant associations between *VDR* SNVs and the outcomes, atopy, asthma or asthma severity. Regarding atopy, 792 individuals were included, 364 cases and 428 controls. Allele (A) of SNV rs10875694 located in an intronic region of the gene was more frequent on atopic individuals and positively associated with atopy OR = 1.35 (95% CI 1.01–1.81; p-value = 0.043). In the analysis for asthma, 63 cases were included (all asthma cases of the study) and 729 controls. The allele C of variant rs9279, a 3-UTR-prime, was associated with lower risk of asthma OR = 0.66 (95% CI 0.45–0.97; p-value = 0.033). Regarding asthma severity 38 severe asthma cases and 25 mild/moderate asthma controls were included. The variants in *VDR* showed to be associated were the rs2189480 and the rs4328262, both placed on the intronic region. The first variant was negatively associated (OR = 0.34; 95% CI 0.13–0.89; p-value = 0.029) and the second one was positively (OR = 3.18; 95% CI 1.09–9.28; p-value = 0.034) associated with severe asthma.

**Table 2:** Significant associations between SNVs in *VDR* gene with atopy, asthma symptoms and asthma severity

Phenotype	SNVs	Alele	Model	OR^a^	CI 95%	*p* value^b^
Atopy^c^						
*VDR*	rs10875694	A	ADD	1.35	1.01–1.81	0.043
Asthma symptoms						
*VDR*	rs9729	C	ADD	0.66	0.45–0.97	0.033
Severe asthma						
*VDR*	rs2189480	A	ADD	0.34	0.13–0.89	0.029
	rs4328262	G	ADD	3.18	1.09–9.28	0.034

^a^Using logistic regression adjusted by sex, age, and individual genetic ancestry

^b^Permutational analysis

^c^sIgE ≥ 0.70 to common aeroallergens; *ADD* additive analysis model

### SNVs on Vitamin D pathway (*CYP2R1*, *CYP24A1* and *VDR*) were associated with vitamin D levels

Table 3 shows the significant associations on SNVs of vitamin D pathway genes with different classifications of vitamin D levels in serum. Using logistic regression, we found negative associations for vitamin D insufficiency and *VDR* gene for 6 SNV’s (rs7967152, rs9729, rs739837, rs11168287, rs7963776 and rs4237855) and in *CYP24A1* for 2 SNVs (rs4809960 and rs2245153), which means that the presence of above variants reduce the possibility of a certain individual to be insufficient for 25(OH)D serum levels. While positive associations were found for 3 SNVs in *VDR* (rs59128934, rs7965274 and rs2853564), 2 SNVs in *CYP24A1* (rs56229249 and rs34043203) and 2 in *CYP2R1* (rs12794714 and rs10500804), indicating that the presence of such variations increase risk to vitamin D insufficiency. One SNV (rs59128934) on *VDR* and another (rs3886163) on *CYP24A1* were associated with increased risk to Vitamin D deficiency. When we assessed continuous levels of serum 25(OH)D, two SNVs in *CYP2R1* (rs12794714 and rs105008804) and one in *CYP24A1* were associated with a lower levels of 25(OH)D in serum.

**Table 3:** Significant associations between SNVs in *VDR, CYP2R1, CYP24A1,* vitamin D status and 25(OH)D serum levels

Phenotype	SNVs	Alele	Model	OR^a^	CI 95%	P value
25(OH)D serum levels						
		Alele	Model	*B*^c^	CI 95%	*P value*
*CYP2R1*						
	rs12794714	A	ADD	− 1.38	− 2.40 to 0.35	0.009
	rs10500804	G	ADD	− 1.37	− 2.40 to 0.35	0.009
*CYP24A1*						
	rs3886163	T	ADD	− 1.48	− 2.77 to 0.18	0.026
Vitamin D defficiency						
*VDR*						
	rs59128934	G	ADD	1.78	1.12 to 2.83	0.014
*CYP24A1*						
	rs3886163	T	ADD	1.44	1.05 to 1.99	0.025
Vitamin D insufficiency						
*VDR*						
	rs7967152	A	ADD	0.77	0.62 to 0.95	0.013
	rs9729	C	ADD	0.78	0.70 to 0.96	0.017
	rs739837	G	ADD	0.78	0.63 to 0.96	0.019
	rs11168287	G	ADD	0.78	0.63 to 0.97	0.028
	rs7963776	G	ADD	0.79	0.64 to 0.98	0.029
	rs4237855	G	ADD	0.79	0.63 to 0.99	0.038
	rs59128934	G	ADD	2.07	1.28 to 3.34	0.002
	rs7965274	T	ADD	1.31	1.01 to 1.70	0.044
	rs2853564	C	ADD	1.30	1.00 to 1.70	0.049
*CYP2R1*						
	rs12794714	A	ADD	1.41	1.11 to 1.79	0.005
	rs10500804	G	ADD	1.40	1.11 to 1.77	0.006
*CYP24A1*						
	rs4809960	C	ADD	0.69	0.53 to 0.91	0.008
	rs2245153	C	ADD	0.79	0.63 to 0.99	0.042
	rs56229249	G	ADD	1.42	1.04 to 1.94	0.028
	rs34043203	A	ADD	1.49	1.00 to 2.22	0.049

*ADD* additive analysis model

^a^Using logistic regression adjusted by sex, age and individual genetic ancestry

^b^Permutational analysis

^c^Linear regression B coefficient adjusted by sex, age, helminth infection and individual genetic ancestry

The variant in *VDR* rs59128934 (allele G) was associated with risk to vitamin D insufficiency (OR 2.07; 95% CI 1.28–3.34; p = 0.002) as well as to deficiency (OR 1.78; 95% CI 1.12–2.83; p = 0.014), when compared to controls, ≥ 30 ng/mL and ≥ 20 ng/mL, respectively. The variant rs9729 (allele C), was negatively associated with asthma, and also negatively associated with insufficiency of vitamin D (OR 0.78; 95% CI 0.70–0.96; p = 0.017), which means that the carrier of this variant had a lower possibility to be asthmatic and had insufficient levels of 25(OH)D. The genotypic frequency of the studied SNVs by outcomes are shown in Additional file 1: Table S2.

**Fig. 2:**
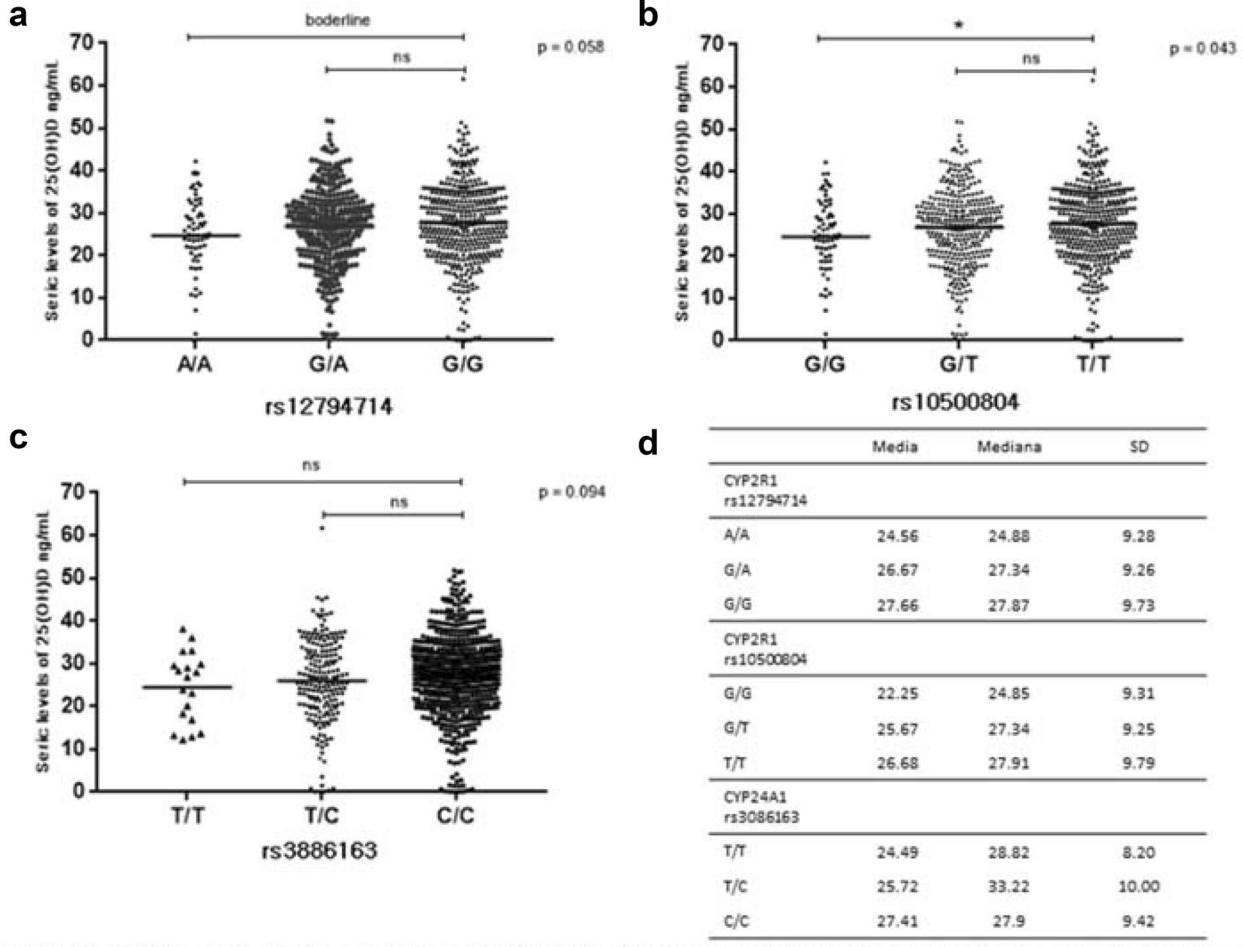
25OH(D) seric levels by genotypes on SNVs in CYP2R1 and CYP24A1. The **a**, **b** and **c** graphic shows the distribution of 25OH(D) seric levels according genotypes to related SNV’s, horizontal bars represent mean values. p values refers Kruskal–Wallis test and superior bar refer Dunn post test. D table represent measures of central tendency and dispersion to each SNV genotype. p = Kruskal–Wallis analysis. *ns* non-significant. *p < 0.05 Dunn post test

The SNVs rs12794714 (allele A) and rs10500804 (allele G) on *CYP2R1* and rs3886163 (allele T) on *CYP24A1* were associated with low levels of vitamin D. To better view the effect of genetic variants on vitamin D levels, we have represented in Fig. 2 the 25(OH)D serum levels by the genotype of the SNVs above mentioned. Carriers of (G) allele of rs10500804 variant on *CYP2R1* have lower 25(OH)D serum levels (Fig. 2b; p < 0.05). The other two variants presented had no statistical significance with 25(OH)D by using Kruskal–Wallis test (Fig. 2a, d).

### Genetic risk score using CYP variants influence 25(OH)D serum levels

**Fig. 3:**
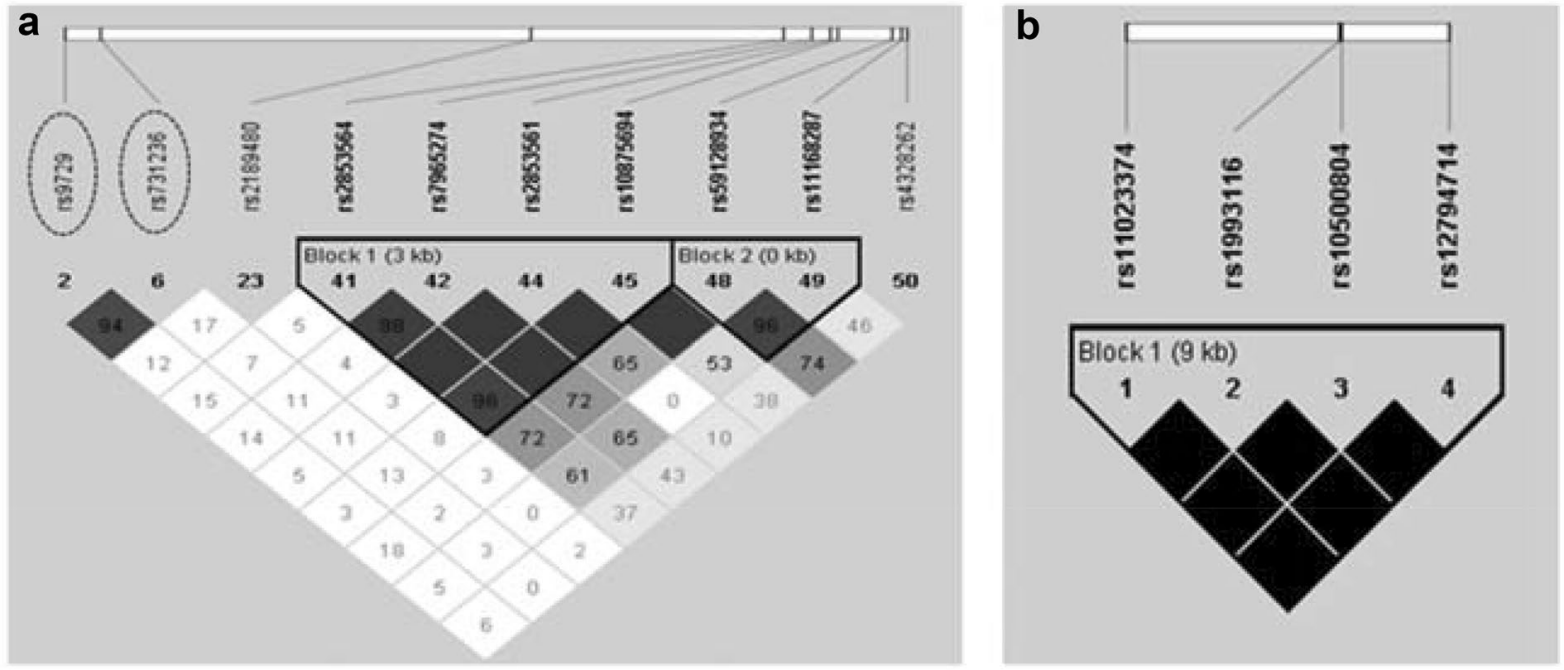
Linkage disequilibrium (r^2^) in VDR gene on SCAALA population. The LD plot was generates by Haploview program using PLINK 1.07 data set. The top horizontal bar illustrates the SNV’s location on physical scale. The squares colour illustrates the strength of pairwise r^2^ values scale, where black indicate perfect LD (r^2^ = 1), grey indicate imperfect LD (1 > r^2^ < 0) and white indicate equilibrium (r^2^ = 0). LD value is also indicate inside each square. In **a** VDR, the rs9729 and rs731236 are in high LD. In **b** CYP2R1, rs10500804 and rs12794714 are in perfect LD

To understand the combined effect of variants on *CYP2R1* and *CYP24A1* we performed a genetic risk score analysis using SNPStats web version (Table 4). Together, both polymorphic alleles on *CYP2R1* (G;A, to rs10500804, rs12794714, respectively) were associated with decreased 25(OH)D serum level (*B* = − 1.24; CI 95% − 2.42 to − 0.06; *p*-*value* = 0.040). The association increased when we added the polymorphic allele on *CYP24A1* (T to rs3886163) in the analysis (*B* = − 3.29; CI 95% − 6.19 to − 0.39; *p*-*value* = 0.027). The *CYP2R1* variants are in complete linkage disequilibrium (Fig. 3).

**Table 4:** Genetic risk score analysis between SNVs in *CYP2R1, CYP24A1* and 25(OH)D serum levels

	*CYP2R1*		*CYP24A1*	25(OH)D serum concentration		
	rs10500804	rs12794714	rs3886163	Frequency	*β (*CI 95%)^a^	*p value
1	T	G	C	0.6016	00	–
2	T	G	T	0.1042	− 1.41 (− 3.25 to 0.42)	0.130
3	G	A	C	0.2513	− *1.24 (*− *2.42* to − *0.06)*	*0.040*
4	G	A	T	0.0422	− *3.29 (*− *6.19* to − *0.39)*	*0.027*

^a^Linear regression B coefficient adjusted by sex, age and individual genetic ancestry

*Snpstat p value

In italic statistically significant association, in italic borderline association

### Variants in *VDR* increase VDR gene expression

**Fig. 4:**
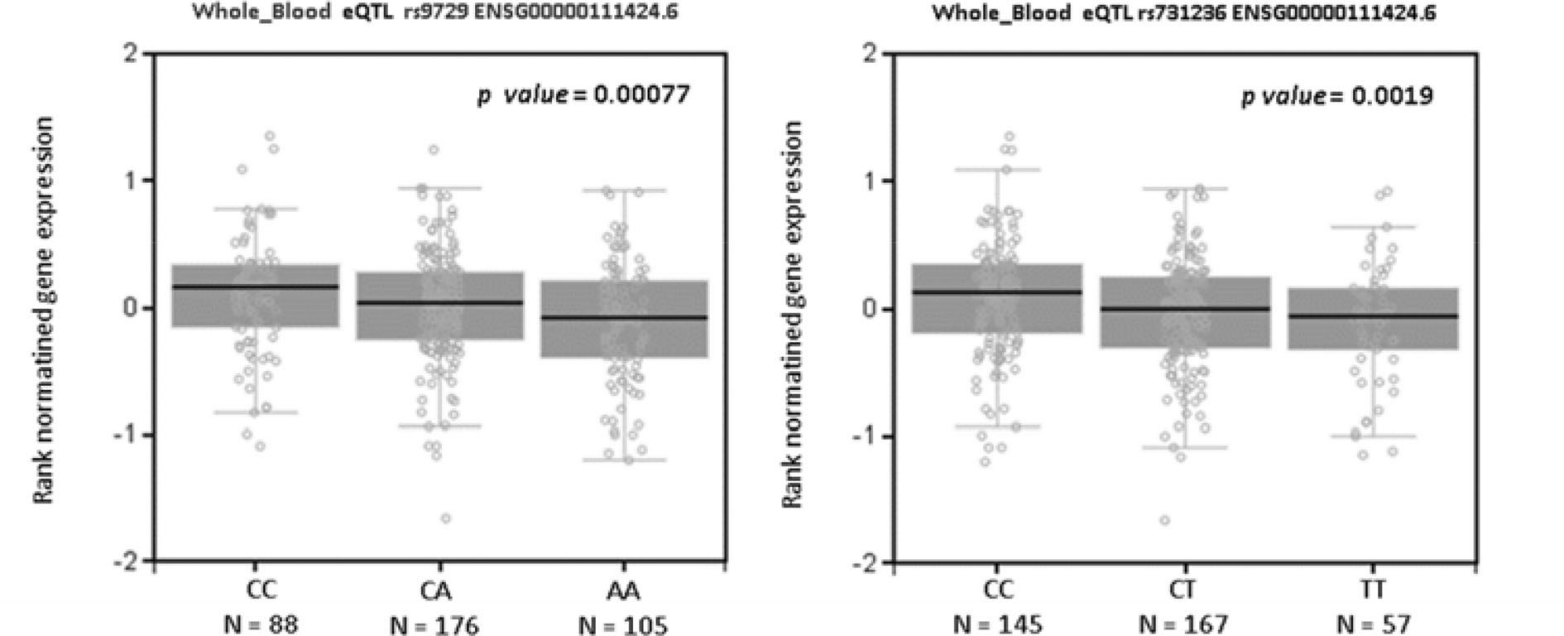
VDR expression on whole bloond to rs9729 and rs731236. The NES is similar to both variants, their reduce VDR expression. *NES* normatined gene expression

To better understand how such variants affect vitamin D activity we checked 25(OH)D distribution by genotypes (data not shown) and checked how it affects *VDR* expression using the online platform GTEx. As can be seen in Fig. 4, the C allele of rs9729 increased *VDR* expression (p-value = 0.0007). This variant (rs9729) is placed in a 3-prime-UTR region and is in high linkage disequilibrium (LD) with other SNV in *VDR,* such as rs731236 (Fig. 3) that is placed on exon 9 a synonymous polymorphism. Therefore, we analysed the variant rs731236 in GTEx and we had similar results to rs9729, the C allele increases *VDR* expression, GTEx p-value = 0.0019 (Fig. 4).

## Discussion

Genetic studies have helped to understand the pathologic pathways of complex diseases such as asthma and other allergic diseases determined by a complex interaction of a variety of genes and environmental factors [39, 60]. There is solid evidence that Vitamin D plays an important biological role in the immune system and affects immune-mediated diseases [61]. However, its role in asthma and allergies is controversial. While most studies have shown protection others have shown no effect [62–64]. Several studies have found single-nucleotide variants in genes of vitamin D pathway associated with asthma and atopy [47, 65, 66].

Vitamin D production is known to be affected by sun exposure [67]. In this way, countries with a higher solar incidence should present population with higher levels of 25(OH)D. Nevertheless, vitamin D insufficiency or deficiency is frequently reported in populations from sunny countries [68, 69]. Therefore, genetic factors could be involved in vitamin D production on such populations.

There are significant reports about SNV’s in vitamin pathway associated with asthma or atopy [48]. The most reported *VDR* SNV’s associated with asthma are, rs1544410 (*Bsm*I), rs7975232 (*Apa*I*)*, rs731236 (*Taq*I) and rs2228570 (*Fok*I) in several populations [42, 48] include Brazilian [43]. In the present study the selected SNV’s was part of a commercial chip contained 2.5 million SNVs (Illumina Human Omni 2.5 BeadChip), that of above mentioned contain only *Bsm*I and *Taq*I SNV’s, however in our population *Bsm*I is out of HWE (p = 0.01), being excluded of analysis and *TaqI* was not directly associated with any outcome.

The present study has been carried out in a population from a city of tropical climate, located in Northeast Brazil, with the average yearly temperature of 27 °C (IBGE), and an estimated average of radiation of 5.9 kWh/m^2^ [49]. However, we found that at least 60% of the studied subjects had insufficient levels of 25(OH)D(Submitted paper).

In our present study, the C allele of variant rs9729 on VDR showed to be negatively associated with asthma and vitamin D insufficiency. This variant was previously reported in a study about allergen sensitization using haplotype analysis [70–72]. The variant rs9729 was poorly explored in asthma and atopy contexts. It is located on 3-UTR-prime, thus it can regulate RNAm stability and *VDR* expression or translation [73]. GTEx in silico gene expression analysis showed that allele C increases *VDR* expression. In our population, this variant is in high linkage disequilibrium (r^2^ = 1) with a synonymous variant widely described in asthma studies, the SNV rs731236 (*TaqI*). The T allele of rs731236 was associated with risk to atopy and asthma [74]. While in Irland, the C allele of rs731236 was associated with risk to uncontrolled asthma [75], 2018).

In the present work, two *VDR* variants were associated with severe asthma, the rs2189480 (allele A) as a protective factor and rs4328262 (allele G) as a risk factor. To our knowledge, it is the first study that reports these associations. In a previous GWAS work, conducted in a paediatrics population elsewhere, these variants were not associated with asthma [76]. The variant rs2189480 allele T was described as a risk factor to melanoma and C allele with protection to Type 2 diabetes [77]. We hypothetized that rs2189480 affect regulatory T cell function, once T help regulate inflammatory activity on asthma [65] and possibility tumour proliferation [66], thus explained opposite association found. Functional study of this variant in T cell was requested to elucidate this question.

The variant rs4328262 (allele G) was described in association with breast cancer reduced risk in North America [78], European and East Asian women [79]. Also, it was described in association with increased visceral adipose tissue [80]. However, again it is the first report of this association on asthma. The functional effect on VDR is not clear yet in the presence of this variant.

Also on VDR, we also found the A allele of rs10875694 is associated with risk to atopy. This is also the first study that describes this association. However, Reimers and collaborators (2015) have described that carriers of A allele presented slightly lower levels of 25(OH)D than T carriers [81]. At this point, we did not observe a difference in vitamin D production according to different alleles of this variant. This variant is placed in an intronic region, however, no functional data analysed here using both RegulomeDB and GTEx, did not shown a possible functional impact of this polymorphism on VDR activity. Although one could consider that being an intronic SNVs they could modify messenger RNA (RNAm) stability and also play a role in translational efficiency [73, 82]. Thus, we can suppose that this genetic variant may affect *VDR* expression, and VDR can, in its turn, control the expression of other genes involved in the development of atopy.

We found three SNV’s on two important enzymes of vitamin D metabolic pathway [rs10500804 and rs12794714 (*CYP2R1*) and rs3886163 (*CYP24A1*)] associated with decreased serum level of vitamin D. Additionally the genetic risk score analysis showed that in the presence of the three alleles all together, the effect in decreasing vitamin D serum levels is even stronger.

CYP2R1 is the principal 25-hydroxylase enzyme of the vitamin D pathway. Mutations on this enzyme that inhibits hydroxylase activity can result in 25(OH)D deficiency [83]. The SNV’s [rs10500804 (G) and rs12794714 (A)] on this gene were associated with lower 25(OH)D concentrations in Arabic population; the homozygote carriers of this SNV in South Asian presented lower levels but there was not statistically significant association in comparison with T and G carrier respectively. [84]. Also, these variants were associated with lower 25(OH)D levels after supplementation with vitamin D in USA [85]. In this way, such variants can affect CYP2R1, reducing its metabolic activities. Further studies are needed in order to address if carriers of these variants may have any benefit of vitamin D intake having a deficiency in vitamin D levels due to CYP2R1 enzymatic alterations.

CYP24A1 is the principal catabolic enzyme in vitamin D pathway; knockout mice to this enzyme are not able to reduce vitamin D levels and its loss causes idiopathic infancy hypercalcemia [86, 87]. Genetic polymorphisms in this enzyme affect vitamin D metabolism, and are associated with cancer and coronary atherosclerosis risks [88]. In a previous study about evaluating the pulmonary function, the allele T of the variant rs3886163 on *CYP24A1* was associated with better forced expiratory volume in 1 s (FEV1), although no association was found with vitamin D serum levels [89]. It was also associated with a lower coronary artery calcification [88]. In the present study, its association with lower 25(OH)D levels suggests that the T allele of rs3886163 possibly increases enzymatic activity. In North American population the variant allele (T) was associated with better pulmonary function and in European it was associated with less calcification in lungs, thus, suggesting that such SNV increases vitamin D levels, once higher levels of vitamin D cause less artery calcification [90] and increased pulmonary function [91]. The opposite results should be explored, once CYP24A1 affects 1,25(OH)_2_D and 25(OH)D levels, and metabolites formed by this enzyme are ten times more frequent than 1,25(OH)_2_D; these metabolites (1,24,25(OH) _3_D or 24,25(OH)_2_D) can have some non-related activity in immunologic cells that could help to understand the vitamin D role in a series of immunological related-diseases. In addition, more functional studies should be performed to understand the impact of this SNV in CYP24A1 activity and in 25(OH)D levels in blood.

Regarding vitamin insufficiency, one SNV in *VDR* (rs59128934 allele G), and one SNV in *CYP24A1* (rs3886163 allele T), were associated with higher risk. The rs59128934 is placed in an intronic region on *VDR* gene, and from our knowledge there was no report about this SNV in any previous study, once more this is the first report of any association with this variant. Here (in this study), although no association was found for rs59128934 with asthma, we hypothesized that since this SNV was able to decrease vitamin D levels, that could lead to asthma. Unfortunately, we were unable to capture this association probably due to the number of asthma cases with detectable levels of vitamin D. The rs3886163 T allele was reportedly associated with better FEV1 in American Africans, but their effect on vitamin D levels was not demonstrated [89].

Six variants in *VDR* gene were associated reducing the risk of insufficiency, rs7967152 (A), rs9729(C), rs739837(G), rs11168287(G), rs7963776(G), rs4237855(G). While three other variants were associated with increased risk of insufficiency rs59128934(G), rs7965274(T) and rs2853564(C). There are few studies showing a relationship between *VDR* polymorphism and 25(OH)D levels. The VDR regulates vitamin D metabolism, the binding of 1,25(OH)_2_D in VDR, promoting reduced *CYP2R1* and *CYP27B1* gene transcription, and promoting CYP24A1 expression leading reduction of vitamin D levels. It’s a negative feedback mechanism to avoid excessive levels of the active metabolite [92]. Its important to highlight that rs739837 C allele creates a binding site to miRNA-34b in *VDR*, decreasing their expression [93].

The SNV’s in *CYP2R1* rs10500804 and rs12794714 correlated with a lower levels of vitamin D, were also associated with risk to vitamin D insufficiency. The variant rs3886163 in *CYP24A1* correlated with reduction of vitamin D levels also were associated with deficiency. This finding deserves attention once these variants related to lower serum 25(OH)D at the time to be a risk factor to individual health.

### In this work we we had found associations between variants in *VDR* gene and different categories vitamin D levels in blood, however no previous study was found in literature describing such associations before

A limitation of this study was that we did not measure the 1,25(OH)_2_D levels, due to a lack of funding to perform it in all our sample. But there is a plan to do at some moment in the future. This result would allow us to better understand the complete vitamin D metabolism down to its active metabolite.

## Conclusions

In conclusion, we have demonstrated that variants in genes from the vitamin D pathway may affect vitamin D levels and can lead to asthma and atopy. In our population, the vitamin D insufficiency can be due to genetic variations in genes of enzymes that directly influence vitamin D levels. Further studies are necessary, including strategies looking at markers of vitamin D pathway such as PTH and 1,25(OH)_2_D to better understand the impact of genetic variants on the metabolism of vitamin D, including as interaction analysis to understand its role in asthma and atopy.

## Supplementary information

**Additional file 1: Table S1.** Genetic variants in vitamin pathway associated with atopy or asthma, or 25 (OH) D serum levels. **Table S2.** Genotypic Frequency of associates SNV’s in vitamin D pathway by outcomes.

## Data Availability

The data that support the findings of this study are available from the corresponding author on reasonable request.
