# Supplementary material for: Genetic polymorphisms in vitamin D pathway influence 25(OH)D levels and are associated with atopy and asthma
Source: Allergy Asthma Clin Immunol. 2020 Jul 9;16:62. doi: 10.1186/s13223-020-00460-y (PMC7386242; doi:10.1186/s13223-020-00460-y)
Supplement: Supplementary file 1 — Additional file 1: Table S1. Genetic variants in vitamin pathway associated with atopy or asthma, or 25 (OH) D serum levels. Table S2. Genotypic Frequency of associates SNV’s in vitamin D pathway by outcomes. [file 13223_2020_460_MOESM1_ESM.docx]

| **Table S1: Genetic variants in vitamin pathway associated with atopy or asthma, or 25 (OH) D serum levels** | | | | | | |
| --- | --- | --- | --- | --- | --- | --- |
| **Chr** | **SNV** | **Gene** | **Annotation** | **Minor Allele** | **Reference Allele** | **MAF** |
| 12 | rs9729 | *VDR* | 3-prime-UTR | C | A | 0.4381 |
| 12 | rs10875694 | *VDR* | Intron variant | A | T | 0.127 |
| 12 | rs2853561 | *VDR* | Intron variant | C | C | 0.4015 |
| 12 | rs2189480 | *VDR* | Intron variant | A | C | 0.3448 |
| 12 | rs59128934 | *VDR* | Intron variant | G | T | 0.0648 |
| 12 | rs7967152 | *VDR* | Intron variant | A | C | 0.4179 |
| 12 | rs739837 | *VDR* | 3-prime-UTR | G | T | 0.4381 |
| 12 | rs4328262 | *VDR* | Intron variant | G | T | 0.3487 |
| 12 | rs11168287 | *VDR* | Intron variant | G | A | 0.3696 |
| 12 | rs7963776 | *VDR* | Intron variant | G | A | 0.4377 |
| 12 | rs4237855 | *VDR* | Intron variant | G | A | 0.3071 |
| 12 | rs7965274 | *VDR* | Intron variant | T | C | 0.2036 |
| 12 | rs2853564 | *VDR* | Intron variant | C | T | 0.1992 |
| 11 | rs10500804 | *CYP2R1* | 5-prime-UTR | G | T | 0.2869 |
| 11 | rs12794714 | *CYP2R1* | Intron variant | A | G | 0.2852 |
| 20 | rs3886163 | *CYP24A1* | Intron variant | T | C | 0.1371 |
| 20 | rs4809960 | *CYP24A1* | Intron variant | C | T | 0.3434 |
| 20 | rs56229249 | *CYP24A1* | Intron variant | G | A | 0.1317 |
| 20 | rs2245153 | *CYP24A1* | Intron variant | C | T | 0.2569 |
| 20 | rs34043203 | *CYP24A1* | Intron variant | A | G | 0.0817 |

| **Table S2: Genotypic Frequency of associates SNV’s in vitamin D pathway by outcomes** | | | |  |
| --- | --- | --- | --- | --- |
|  | | | |  |
| **Outcomes** | **Genotype** | **Control**  **N (%)** | **Case**  **N (%)** |  |
| **Atopy** |  | **428 (54.0)** | **364 (46.0)** |  |
| ***VDR*** | **rs10875694^*^** |  |  |  |
|  | **TT** | 338 (55.9) | 267 (44.1) |  |
|  | **AT** | 83 (49.7) | 84 (50.3) |  |
|  | **AA** | 7 (38.9) | 11 (61.1) |  |
| **Asthma symptoms** | | **729 (92.0)** | **63 (8.0)** |  |
| ***VDR*** | **rs9729** |  |  |  |
|  | **AA** | 223 (88.9) | 26 (10.4) |  |
|  | **CA** | 350 (92.3) | 29 (7.7) |  |
|  | **CC** | 156 (95.1) | 8 (4.2) |  |
| **Asthma severity** | | **38 (60.3)** | **25 (39.7)** |  |
| ***VDR*** | **rs2189480** |  |  |  |
|  | **CC** | 12 (46.2) | 14 (53.8) |  |
|  | **CA** | 18 (64.3) | 10 (35.7) |  |
|  | **AA** | 8 (88.0) | 1 (11.2) |  |
|  | **rs4328262** |  |  |  |
|  | **TT** | 18 (72.0) | 7 (28.0) |  |
|  | **GT** | 18 (54.2) | 15 (45.5) |  |
|  | **GG** | 2 (40.0) | 3 (60.0) |  |
| **Vitamin D insufficiency** |  | **305 (38.5)** | **487 (61.5)** |  |
| ***VDR*** |  |  |  |  |
|  | **rs7967152** |  |  |  |
|  | **AA** | 60 (42.9) | 80 (57.1) |  |
|  | **AC** | 161 (40.7) | 235 (59.3) |  |
|  | **CC** | 84 (32.8) | 172 (67.2) |  |
|  | **rs9729** |  |  |  |
|  | **AA** | 87 (34.9) | 162 (65.1) |  |
|  | **AC** | 143 (37.7) | 236 (62.3) |  |
|  | **CC** | 75 (45.7) | 89 (54.3) |  |
|  | **rs739837** |  |  |  |
|  | **GG** | 73 (46.8) | 83 (53.2) |  |
|  | **GT** | 149 (37.2) | 251 (62.8) |  |
|  | **TT** | 83 (35.2) | 153 (64.8) |  |
|  | **rs11168287** |  |  |  |
|  | **AA** | 116 (35.0) | 215 (65.0) |  |
|  | **GA** | 148 (39.9) | 223 (60.1) |  |
|  | **GG** | 41 (45.6) | 49 (54.4) |  |
|  | **rs7963776** |  |  |  |
|  | **AA** | 80 (33.2) | 161 (66.8) |  |
|  | **GA** | 161 (40.6) | 236 (59.4) |  |
|  | **GG** | 64 (41.6) | 90 (58.4) |  |
|  | **rs4237855** |  |  |  |
|  | **AA** | 143 (35.8) | 256 (64.2) |  |
|  | **GA** | 127 (39.6) | 194 (60.4) |  |
|  | **GG** | 35 (48.6) | 37 (51.4) |  |
|  | **rs59128934^**^** |  |  |  |
|  | **GG** | 0 | 1 (100.0) |  |
|  | **GT** | 25 (25.0) | 75 (75.0) |  |
|  | **TT** | 280 (40.7) | 408 (59.3) |  |
|  | **rs7965274**^***^ |  |  |  |
|  | **CC** | 203 (40.4) | 297 (59.4) |  |
|  | **CT** | 94 (36.2) | 165 (63.8) |  |
|  | **TT** | 8 (25.0) | 24 (75.0) |  |
|  | **rs2853564^#^** |  |  |  |
|  | **AA** | 7 (33.3) | 23 (76.7) |  |
|  | **AT** | 93 (36.6) | 161 (63.4) |  |
|  | **TT** | 203 (40.3) | 301 (59.7) |  |
| ***CYP2R1*** |  |  |  |  |
|  | **rs10500804** |  |  |  |
|  | **GG** | 17 (25.8) | 49 (74.2) |  |
|  | **GT** | 122 36.5) | 212 (63.5) |  |
|  | **TT** | 166 (42.3) | 226 (57.7) |  |
|  | **rs12794714^##^** |  |  |  |
|  | **AA** | 16 (25.5) | 47 (74.5) |  |
|  | **AG** | 121 (36.4) | 211 (63.6) |  |
|  | **GG** | 164 (42.3) | 224 (57.7) |  |
| ***CYP24A1*** |  |  |  |  |
|  | **rs4809960^*^** |  |  |  |
|  | **CC** | 11 (44.0) | 14 (56.0) |  |
|  | **CT** | 94 (46.1) | 110 (53.9) |  |
|  | **TT** | 200 (35.7) | 361 (64.3) |  |
|  | **rs2245153***** |  |  |  |
|  | **CC** | 26 (45.6) | 31 (54.4) |  |
|  | **CT** | 121 (41.3) | 172 (58.7) |  |
|  | **TT** | 158 (35.8) | 283 (64.2) |  |
|  | **rs56229249^###^** |  |  |  |
|  | **AA** | 241 (40.8) | 349 (59.2) |  |
|  | **AG** | 57 (31.3) | 125 (68.7) |  |
|  | **GG** | 5 (29.4) | 12 (70.6) |  |
|  | **rs34043203** |  |  |  |
|  | **AA** | 2 (28.6) | 5 (71.4) |  |
|  | **AG** | 34 (30.1) | 79 (69.9) |  |
|  | **GG** | 269 (40.0) | 403 (60.0) |  |
| **Vitamin D deficiency** |  | **627 (79.2)** | **165 (20.8)** |  |
| ***VDR*** |  |  |  |  |
|  | **rs59128934^+^** |  |  |  |
|  | **GG** | 1 (100.0) | 0 (0.0) |  |
|  | **GT** | 69 (69.0) | 31 (31.0) |  |
|  | **TT** | 554 (80.5) | 134 (19.5) |  |
| ***CYP24A1*** |  |  |  |  |
|  | **rs3886163** |  |  |  |
|  | **CC** | 469 (81.0) | 110 (19.0) |  |
|  | **TC** | 145 (74.7) | 49 (25.3) |  |
|  | **TT** | 13 (68.4) | 6 (31.6) |  |
| **^*^ 2 cases had not genotype for this variant**  **^**^3 cases had not genotype for this variant**  *****1 case had not genotype for this variant**  **^#^2 cases and 2 control had not genotype for this variant**  **^##^5 cases and 4 control had not genotype for this variant**  **^###^2 controls and 1 case had not genotype for this variant**  **^+^ 1 case and 1 control had not genotype for this variant** | | | |  |
